# Topographical anatomy of the annulus of Zinn

**DOI:** 10.1038/s41598-022-05178-y

**Published:** 2022-01-20

**Authors:** Hester Lacey, Huw Oliphant, Claire Smith, Michael Koenig, Saul Rajak

**Affiliations:** 1 University Hospitals Sussex NHS Trust, Audrey Emerton Building, Eastern Road, Brighton, BN2 0AE UK; 2 University Hospitals Sussex NHS Trust, The Sussex Eye Hospital, Eastern Road, Brighton, UK; 3 Brighton and Sussex Medical School, Medical School Teaching Building, University of Sussex, Brighton, BN1 9PX UK; 4 Department of Cellular Pathology, Royal Sussex County Hospital, University Hospitals Sussex NHS Trust, Eastern Road, Brighton, BN2 5BE UK

**Keywords:** Anatomy, Diseases, Health care, Medical research

## Abstract

The anatomy and even existence of a common tendinous origin of the extraocular eye muscles, or annulus of Zinn, has widely been debated in anatomical literature. This study explored the anatomical origins of the recti muscles, their course into the orbit and the dural connections of the common tendinous origin with the skull base. Twenty orbits of ten adult human cadavers were dissected. The orbital apex and its dural connections were photographed. Histological examination of apical specimens was performed. In all cadavers, extraocular muscles were observed to have a common tendinous origin at the orbital apex, continuous with dural connections extending into the skull base. Accessory slips of the medial rectus were observed across all cadavers. Dual heads of the lateral rectus were observed in fourteen orbits of seven cadavers. The origin of the levator palpebrae superioris appeared to be contiguous with the superior rectus at the common tendinous origin in all but one cadaver. These results support the existence of a common tendinous origin of the extraocular muscles, that is continuous with the skull base dura. In addition, they support the existence of variations in orbital anatomy including dual or accessory muscle slips of the extraocular muscles.

## Introduction

The Annulus of Zinn is described as the common tendinous origin of the four extraocular (EO) rectus muscles, located in the orbital apex. The common tendinous origin is attached around the superior, inferior and medial margins of the optic canal, and laterally around the mid-portion of the superior orbital fissure (SOF), to form a ring like structure from which the rectus muscles arise^1^.

Originally described by Johann Gottfried Zinn in 1755, the common tendinous origin was thought to be formed from uniting of the dura mater covering the cranial fossa and cavernous sinuses with the dura mater of the superior orbital fissure and periorbita within the orbital apex, and is continuous with the dural sheath of the optic nerve^2,3^. The rectus muscles are thought to arise from the anterior part of this common tendinous origin, forming a muscular cone that extend from the apex to attach to the superior, inferior, medial and lateral aspects of the eye, to control voluntary eye muscle movement^1^.

**Figure 1:**
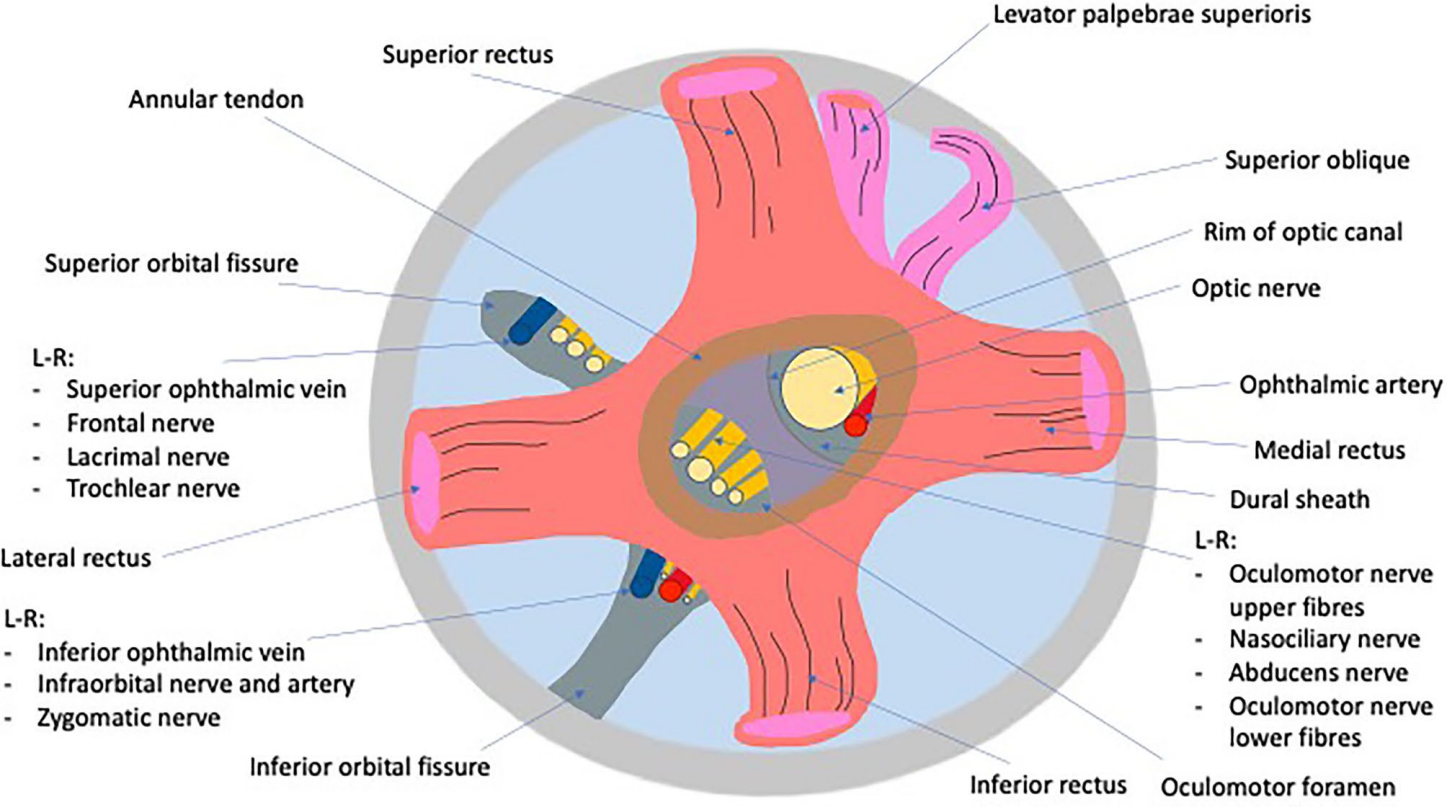
Diagrammatic representation of the anatomy of the orbital apex.

Several neurovascular structures pass through the common tendinous origin. After passing through the optic canal and exiting via the optic foramen, the optic nerve and ophthalmic artery traverse through the common tendinous origin to enter the orbital apex. Via its lateral attachment to the SOF, the tendon divides the SOF into three parts, with the mid part forming the oculomotor foramen, and providing the entry point into the orbit for a number of cranial nerves (CN), including the superior and inferior division of CN III, the nasociliary branch of CN V, CN VI, and the parasympathetic and sensory fibres of the ciliary ganglion^1,4^(Fig. 1).

There are varying and conflicting descriptions in the scientific literature of the anatomy of the common tendinous origin. Some reports describe the common tendinous origin as being separated into two parts; (1) an upper tendon of Lockwood, from which arises the superior rectus (SR), upper parts of the medial rectus (MR), and an upper head of the lateral rectus (LR) and (2) the lower part of the ring which is described as the tendon of Zinn, consisting of the inferior rectus (IR), the remaining parts of the MR, and a lower head of the LR^5^. Dual heads of the rectus muscles are variably described. On the lateral rectus, some studies report this as a consistent finding, and others rare and occasional papers describe the presence of an additional head of the MR, as well as accessory rectus muscles that do not appear to originate from any of the existing rectus muscles^6–8^ The presence of a common tendinous origin of the four rectus muscles has in itself been questioned in some studies, with an independent origin of the SR identified in fetal studies^6,9^.

This study aims to explore the anatomy of the orbital apex using macroscopic and histological examination to further clarify the origins of the rectus muscles and their dural connections and ascertain if they have a common tendinous origin.

## Materials and methods

### Specimens

This study was performed in accordance with the Human Tissue Authority (HTA) guidelines on the handling of human material. Any tissue removed from specimens was disposed of appropriately and with respect to the dignity of the donors. This research has followed the Tenets of the Declaration of Helsinki.

Twenty orbits of ten human cadavers were examined in this study. These cadavers were preserved using a mix of Ethanol (75%), Phenol (5%) and Formaldehyde (3.5%), with up to 20L used for preservation of the largest cadavers. These cadavers were donated to Brighton and Sussex Medical School and their storage and use in their study was approved under the auspices of the Anatomy HTA licence by the Designated Individual at the school responsible for the handling of cadaveric material. Requirements for storage under this license includes consent for use in future research projects. Explicit permission was granted for their use in this study, including the approval of the experimental protocol and the taking of cadaveric images. NHS Research Ethics Committee and Health Research Authority approval was granted for this study.

Cadavers used had the head isolated from the body and the brains had been removed. The dura of the cranium was dissected, and frontal bone was removed over the orbital cavity. Orbital fat was removed to expose the muscles within the orbit. After examination of the origin of the LPS and SR muscle, these were cut distal to their origin to allow for deeper exploration of the orbit. Reflection of the LPS, SR, and ophthalmic artery as well as removal of the anterior section of the optic nerve allowed for visualisation of the remaining EO muscles. Further removal of orbital fat allowed for isolation of the EO muscles. Exploration of the orbital apex and muscle attachments was done and photographed to establish the anatomy and origins of the EO muscles.

From seven donors, one eye (with attached optic nerve and orbital muscles) was examined further in the Department of Cellular Pathology at the Brighton Royal Sussex County Hospital. Six tissue blocks per eye were submitted for histologic study. They were centred around the common tendinous origin with three blocks taken proximal and three distal to the centre of this landmark. Anonymised photographs of relevant histologic areas of one donor were produced and stored. Wax blocks and glass slides were subsequently returned to the donor bodies.

## Results

### Common tendinous ring

**Figure 2:**
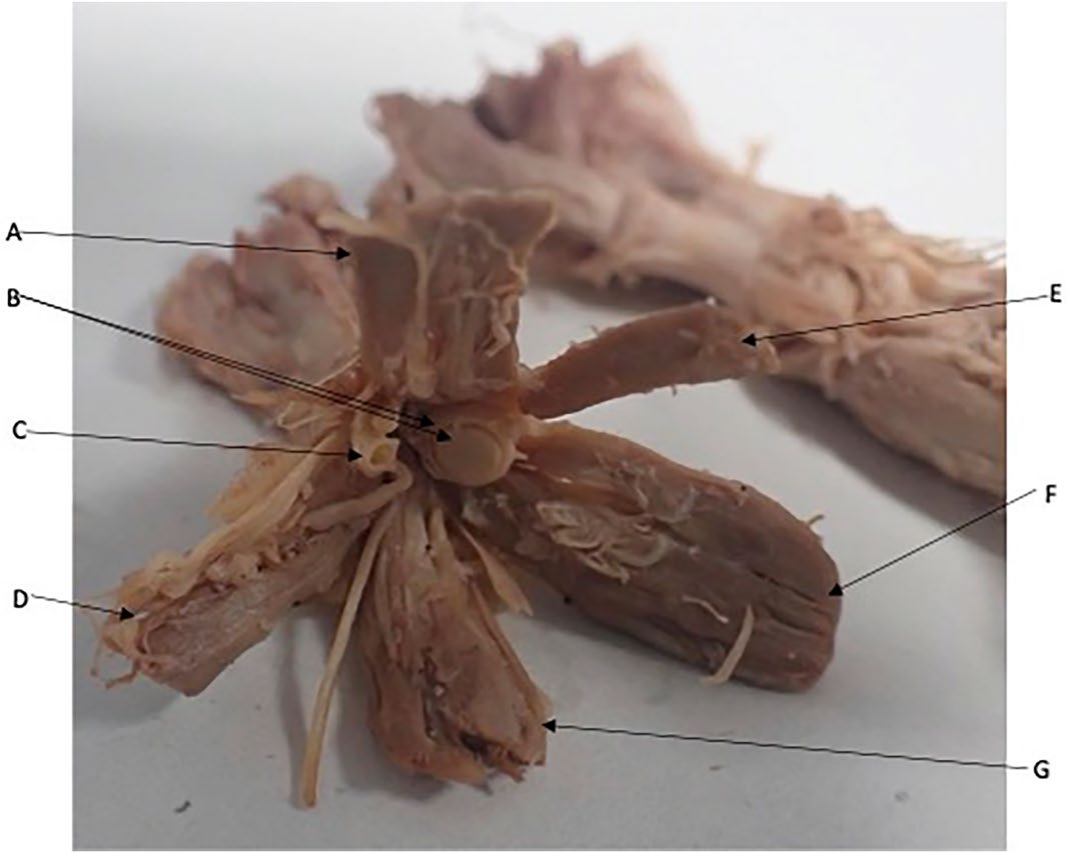
Anterior view of the extraocular muscles extending from a common tendinous origin. Optic nerve (B) containing within dural sheath and coursing through the common tendinous ring. Superior Rectus (A), Lateral Rectus (D), Levator Palpebrae Superioris, Medial Rectus (F), Inferior Rectus (G). Intra-orbital vessels (C).

**Figure 3:**
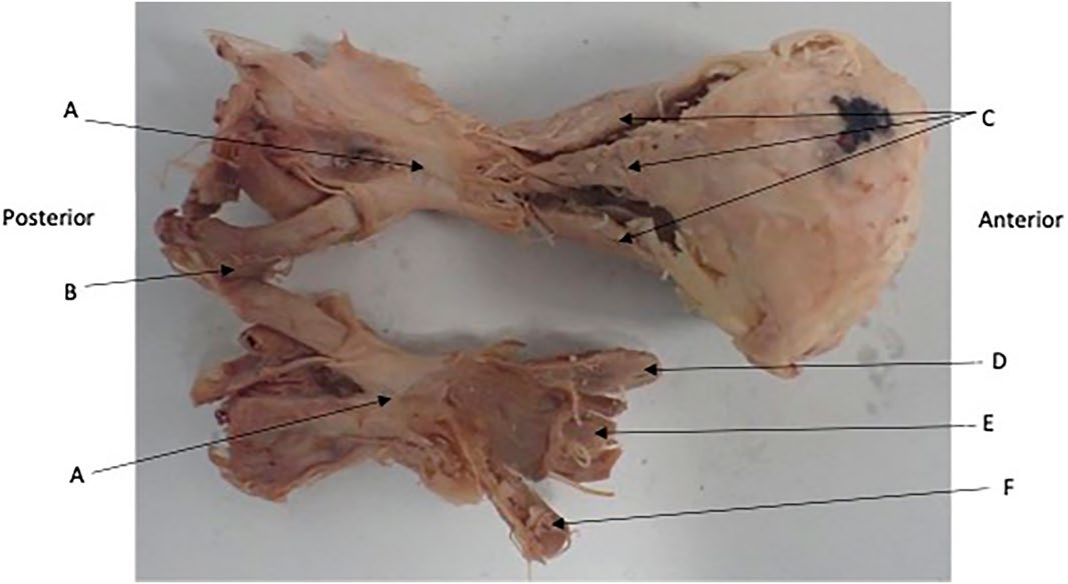
Common tendinous origin (A). Optic Chiasm (B) with bilateral optic nerves extending from it. Muscular cone extending into orbit (C) top to bottom Lateral Rectus, Superior Rectus, Medial Rectus. (D) Levator Palpebrae Superioris, (E) superior rectus, (F) Inferior Rectus.

The EO muscle fibres coalesce to form a common tendinous origin in both sides of all cadavers (Figs. 2,3). This was observed bilaterally in all cadavers dissected. The common tendinous origin is a tubular structure that has an almost sheath like appearance of approximately 10 mm in length before it imperceptibly merges into the extraocular muscles and the dura at either end.

### Histology of the common tendinous ring/common annular tendon (annulus of Zinn)

**Figure 4:**
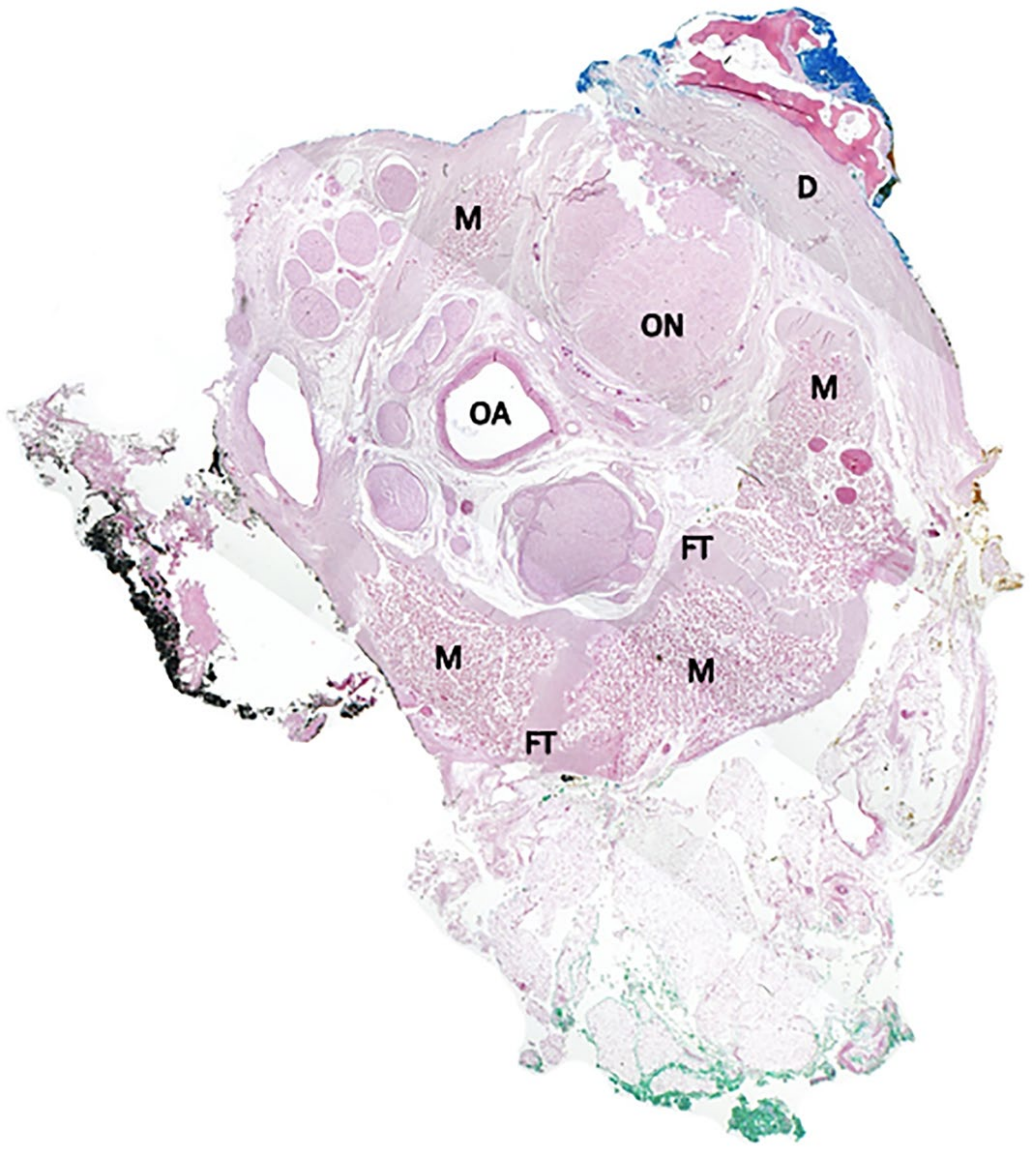
Composite image of histological slides, right orbit. Optic nerve (ON), the ophthalmic artery (OA) is surrounded by oculomotor foramen structures (abducens nerve, nasociliaris nerve, two branches of oculomotor nerve), the skeletal muscle fibres of the extraocular muscles (M) are separated by thin layers of fibrous tissue (FT), the annular fibrous tissue (annulus of Zinn) is continuous with the skull base dura (D).

The sections taken from all seven specimens showed very similar histologic features (Fig. 4). In all sections, the extraocular skeletal muscles fibres gradually merge into fibrous tissue which surrounds the optic nerve and associated structures thus corresponding to the common tendinous ring. The dural sheath surrounding the optic nerve is seen as continuous with this annular fibrous tissue with no separate tissue planes identifiable in any of the specimens. Thus, there is no clear separation between annulus and dura in the specimens examined. The structures encircled by the fibrous ring included the optic nerve, the ophthalmic artery, and four smaller nerves (abducens nerve, nasociliaris nerve and two branches of the oculomotor nerve) as well as some filling fat as suggested by the anatomical literature. Of further interest, in all seven specimens examined, the fibres of each extraocular muscle did not coalesce with other skeletal muscle fibres in the annulus, but they remained separated by thin areas of fibrous tissue (which measured down to 0.2 mm in width).

### Dual heads of the medial rectus

**Figure 5:**
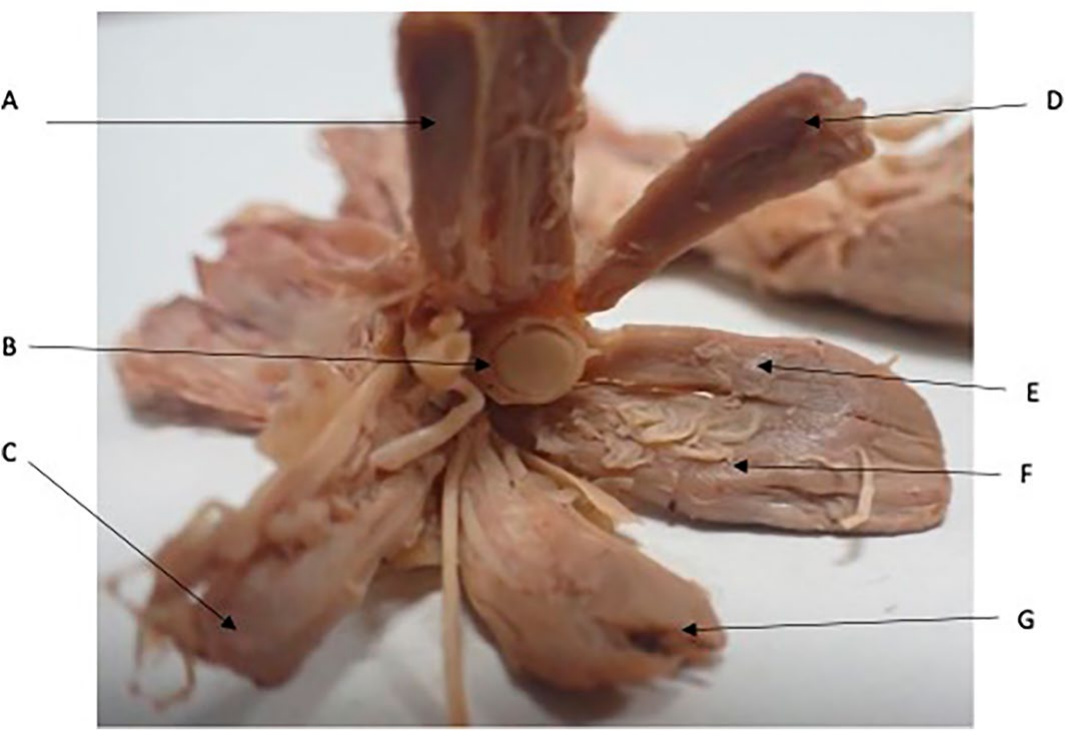
Major (F) and Minor (E) head of the medial rectus. (A) Superior rectus, (B) Optic nerve with surrounding dural sheath, (C) Lateral Rectus, (D) Levator Palpebrae superioris, (G) inferior rectus.

An accessory head of the MR was also visualised in all 20 orbits. The accessory, or minor head was smaller and superior to a larger major head in all cadavers. Two muscles originated from the common tendinous origin before uniting to insert into the medial aspect of the eye. Head were enclosed within the same fascial sheath. (Fig. 5).

### Dual heads of the lateral rectus

**Figure 6:**
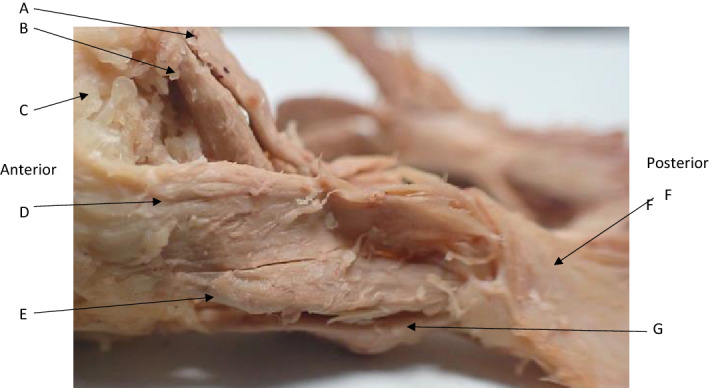
Dual heads of the lateral rectus. Upper head (D) and lower head (E). (A) Superior rectus, (B) Levator Palpebrae superioris, (C) eye, (F) common tendinous origin, (G) Inferior Rectus.

Dual heads of the lateral rectus were observed in fourteen orbits of seven of the cadavers. The inferior head was marginally (1-2 mm) smaller that the superior head. Both heads extended from the common tendinous origin before uniting to insert on the lateral aspect of the eye. Heads were enclosed within the same fascial sheath. (Fig. 6).

### Dural connections

**Figure 7:**
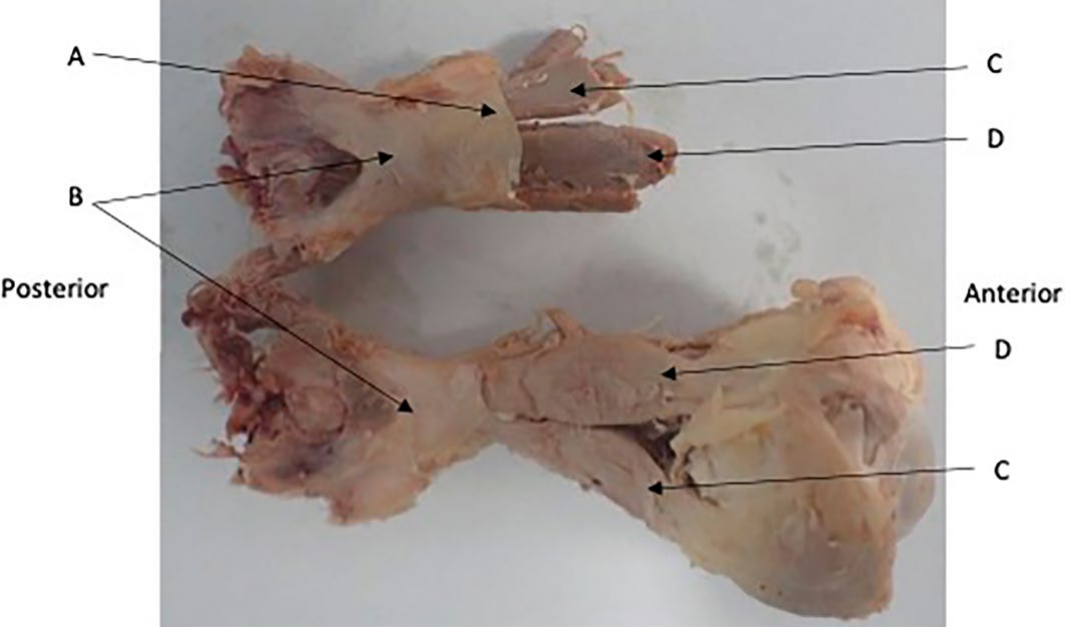
Dural Sheath (A) shown continuing into the orbit anteriorly and posteriorly to unite with the common tendinous origin. Lateral Rectus (C), Inferior Rectus (D).

An examination of the common tendinous ring provided evidence that skull dura was contiguous with the common tendon. The dura of the skull base extended into the orbital apex. Macroscopically it appears to be contiguous with the common origin and enclosed the optic nerve completely through its course through the common tendon and into the orbit. (Figs. 3, 7) This finding was supported by the histologic assessment (above).

### Origins of the superior rectus and levator palpebrae superioris

The SR was observed to originate from the common tendinous ring at the orbital apex (Fig. 2). In 18 orbits of 9 cadavers the LPS was observed coursing alongside the SR, appearing to merge with it at the common tendinous origin (Figs. 2, 3, 5). In the remaining cadaver it was positioned superiorly to the SR, as is commonly discussed in the literature. The LPS could be confirm as a separate muscle to the SR rather than a slip or accessory head as it was enclosed within its own epimysium.

## Discussion

**Figure 8:**
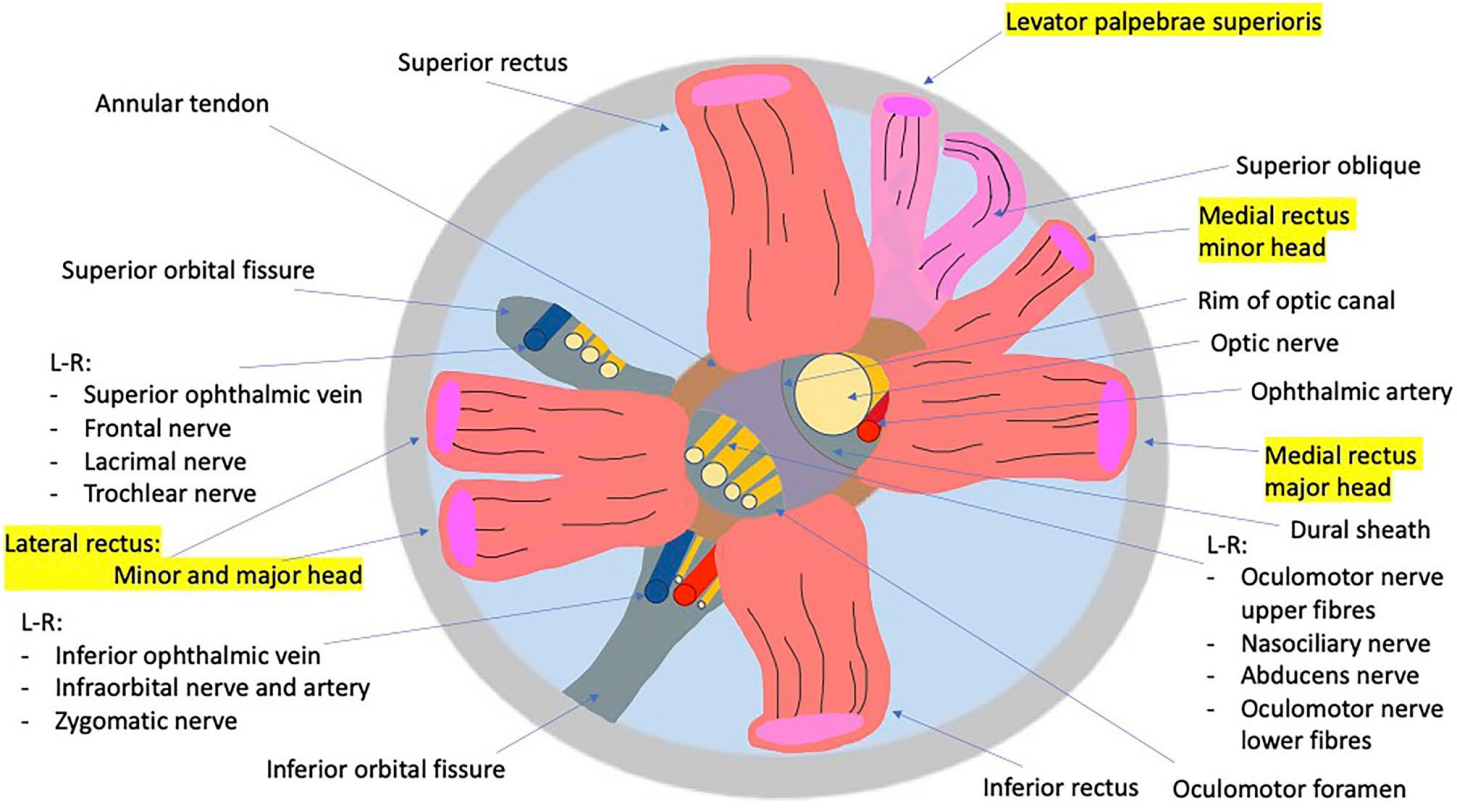
Changes in orbital anatomy observed during dissection compared to original hypothesis.

This study found several conclusions regarding the anatomy of the orbital apex which are summarised in Fig. 8.

There is a paucity of literature on the detailed anatomy of the origins of the recti muscle and even the terminology of this area is confusing with common tendinous origin, tendinous ring, common tendinous ring, annulus of Zinn, annular tendon and annular ligament used variably and interchangeably^10^.

In all cadavers dissected in this study, the four extraocular muscles were seen deriving from a single common tendinous origin at the orbital apex. Macroscopically this origin was tendinous in nature and continuous with the skull base dura. This observation was supported by microscopic assessment which failed to identify a separating anatomical plane between these fibrous tissues. The common tendinous origin had the appearance of an approximately 10 mm tubular structure or ‘tunnel’ rather than a ‘ring’ as may be indicated by its alternative name ‘the annulus of Zinn’. The existence of the common tendinous origin corroborates the results of histological examination obtained in studies by Kim et al. and Naito et al. In our histological examination we found that the tendinous origin is continuous with the skeletal muscle fibres and thus represents their origin. with no separate fibrous layer around them.

In the present study we did not identify any macroscopic or microscopic evidence of the division of the common tendinous origin into two tendons (upper of Lockwood and lower of Zinn) or even three tendons (inferior, inner and external tendons providing the origin for the inferior, medial and external band respectively) as has been described in some studies^6,11,12^ It is possible that they are present in foetuses or younger individuals in keeping with their embryological origin but disappear with the development of skull and facial anatomy that occur with both age and racial variation. This is supported by the histological studies of Naito et al., and Kim et al., whose analysis of foetal specimens described the SR originating alongside the LPS and SO at the superior edge of the optic canal, and the common tendinous origin as the origin for the LR, MR and IR^6,9^.

Accessory heads of medial rectus are described in some studies in the literature, but were present in all orbits in the present study^7,8^. In all orbits, the smaller ‘accessory’ head arose from and ran just superior to the main muscle belly before merging into a single muscle. This concurs with Kim et al., whose embryological histological studies described the accessory head of the MR to originating from the superomedial margin of the optic foramen, adjacent to the SR origin and running laterally to the origin of the SO^6^. While the accessory heads were present as distinct slips in this study, differentiating them from accidental splits in the muscle or from manual separation, heads were enclosed within the same fascial sheath, and inserted adjacent onto the sclera, highlighting their relationship.

The present study additionally suggests that a second head of lateral rectus – which was present in fourteen orbits of seven heads – is not a rare variant as previously indicated in the literature^6,13^ Moreover, in several cadavers, was nearly as large as the major head, effectively bisecting the muscle into two slips. These heads were present as distinct bundles, as with the accessory head of the medial rectus, inserting adjacent to one another onto the sclera and enclosed within the same fascial sheath.

The common tendinous origin was observed macroscopically to be continuous with the dura which was confirmed by histological examination, with no separate tissue planes to see. This is subtly but not substantially different from the description of Zampieri et al. (2015), which describes the common tendinous origin as ‘strictly adherent’ to the dural sheath of the orbital apex^3^.

The origin of LPS is described in the literature as arising from the lesser wing of the sphenoid bone, just superior to the optic foramen^14– 16^. In this study, its origin was difficult to distinguish as separate from that of the superior rectus muscle, arising just above the SR, giving the appearance of a joint origin. Given the shared embryological origin of LPS and SR, it would seem more likely that LPS comes from the common tendinous origin than the lesser wing of sphenoid, although it may have additional muscle fibres extending posteriorly visible on histological examination, similarly to its anterior end which inserts into the tarsal plate as well as the deep surface of the skin at the skin crease^17^. What is apparent from gross cadaveric dissection is that the course of the muscle is contiguous with a common tendinous origin and the origins of the other extraocular muscles. Moreover, the one case in which the LPS was seen originating laterally to the SR, highlights the anatomical variability of the orbital apex and supports it being an entirely separate muscle with its own origin.

The precise anatomy and in particular the composition and thickness of a common tendinous origin and its dural attachments has clinical relevance for orbital surgery. Procedures such as orbital decompression surgery involves removal of bones from the medial, inferior, or lateral portion of the orbit, including at the orbital apex, to relieve pressure in the orbit for conditions such as sight threatening thyroid eye disease. This is particularly relevant in deep (orbital apex) medial wall decompression (undertaken for optic nerve compression in thyroid eye disease) where the common tendinous origin and the optic nerve within it are at risk of traumatic damage from both the instruments – including high power drill – and vasoconstricting drugs such as cocaine and adrenaline that are used during the procedure^18^. The present study demonstrated that the common tendinous ring is comprised of the muscle insertions themselves, and without any additional fibrous protective ring or capsule and therefore damage may be one cause of the diplopia (double vision) that frequently occurs after decompression surgery. Moreover, the common tendinous ring is approximately only a millimetre thick at the apex, and indirect damage to the optic nerve may explain the anecdotal (unpublished, personal communication) reports of visual loss post deep medial wall decompression surgery.

The dural connections also have potential clinical implications in being a possible explanation for autophony, or audible eye movements, in semi-circular canal dehiscence syndrome, in which patients can hear their own eye movements, indicating some sort of anatomical connection between the inner ear and the orbital apex^19,20^.

This was a predominantly macroscopic study, and a surgical microscope and further detailed histological examination would be required to examine the secondary heads of EO muscles to ascertain if they have their own muscular capsule or are present within the same epimysium (2).

This study adds to the relative paucity of literature on the orbital apex. The findings support the existence of a common tendinous origin of the EO muscles that is continuous with the dura of the skull base and indicates that secondary heads of some of the extraocular muscles may be a common finding, highlighting the anatomical variation that can occur in this region.
